# KM04416 suppressed lung adenocarcinoma progression by promoting immune infiltration

**DOI:** 10.1186/s13019-024-02971-w

**Published:** 2024-07-25

**Authors:** Yalan Lin, Weijing Wu, Huihuang Lin, Shiyuan Chen, Huiying Lv, Shuchao Chen, Chuzhao Li, Xinwen Wang, Yunfeng Chen

**Affiliations:** 1 Department of Pulmonary and Critical Care Medicine, Respiratory Medicine Center of Fujian Province, The Second Affiliated Hospital of Fujian Medical University, Quanzhou, China; 2 Department of Oncology, Dongguan People ’s Hospital, Dongguan, China; 3 Department of Orthopedics, Sanming First Hospital Affiliated to Fujian Medical University, Sanming, Fujian China

**Keywords:** KM04416, Lung adenocarcinoma, Progression, Immune infiltration

## Abstract

**Objectives:**

Lung adenocarcinoma (LUAD) is a malignant tumor originating from the bronchial mucosa or glands of the lung, with the fastest increasing morbidity and mortality. Therefore, the prognosis of lung cancer remains poor. Glycerol-3-phosphate dehydrogenase 2 (GPD2) is a widely existing protein pattern sequence in biology and is closely related to tumor progression. The therapy values of GPD2 inhibitor in LUAD were unclear. Therefore, we aimed to analyze the therapy values of GPD2 inhibitor in LUAD.

**Materials and methods:**

The Cancer Genome Atlas (TCGA)-LUAD database was used to analyze the expression levels of GPD2 in LUAD tissues. The relationship between GPD2 expression and LUAD patient survival was analyzed by Kaplan-Meier method. Moreover, KM04416 as a target inhibitor of GPD2 was used to further investigate the therapy value of GPD2 inhibitor in LUAD cells lines (A549 cell and H1299 cell). The TISIDB website was used to investigate the associations between GPD2 expression and immune cell infiltration in LUAD.

**Results:**

The results showed that GPD2 is overexpressed in LUAD tissues and significantly associated with poor survival. KM04416 can suppress the progression of LUAD cells by targeting GPD2. Low expression of GPD2 is related to high infiltration of immune cells.

**Conclusions:**

In summary, our present study found that targeting inhibition of GPD2 by KM04416 can suppress LUAD progression via adjusting immune cell infiltration.

## Introduction

Lung adenocarcinoma (LUAD) is a malignant respiratory system tumor that seriously endangers human health and ranks among the highest incidence and mortality of cancer worldwide [1–3]. The early status of LUAD is complex, the disease progresses rapidly, and the prognosis is poor. Despite the progress in the treatment and survival of LUAD in the past 30 years, the improvement in the survival outcome of LUAD patients is relatively small, and it is still necessary to explore new models for LUAD treatment [4, 5]. Presently, gene-targeted therapy and immunotherapy have progressed, but early diagnosis and treatment are essential for LUAD. Therefore, exploring new potential therapeutic targets can effectively improve the poor prognosis of patients is necessary.

Glycerol-3-phosphate dehydrogenase 2 (GPD2), a type of enzyme embedded into the outer surface of the inner mitochondrial membrane [6], which plays an essential role in glycolysis, fatty acid metabolism, and intracellular oxidative phosphorylation [7], and can be potent inhibited by KM04416, an isothiazolone derivative and potent inhibit glycerol 3-phosphate dehydrogenase (GPD2) [8, 9]. The effects and therapy values of GPD2 in tumor progression have been explored. In prostate cancer cells, study found that GPD2-associated mRNA was higher than in normal prostate epithelial cells and that upregulation of GPD2 promoted an overall increase in ROS production and likely contributed to cancer progression due to enhanced intracellular glycolysis capacity [10]. Thakur et al. [11] found GPD2 as a novel regulator of thyroid cancer growth and metabolism, and suppress GPD2 showed growth-inhibitory effects both in vitro and in vivo in thyroid cancer. The study reported that knockdown the expression of GPD2 in liver cancer cells can disrupt the homeostasis of energy metabolism and decrease cancer development and progression [12]. In a preliminary study, the detections of GPD2 in LUAD and normal tissues in TCGA-LUAD database found that GPD2 is overexpressed in LUAD tissues, and high expression of GPD2 related to poor survival outcome in LUAD patients. Therefore, we hypothesis that GPD2 might have role in LUAD. Our study aims to investigate the status and therapy values of GPD2 in LUAD progression and its related mechanism.

## Materials and methods

### Data and survival analysis

GPD2 expression was evaluated in lung adenocarcinoma and normal tissues in The Cancer Genome Atlas (TCGA) database-LUAD. The Kaplan-Meier (KM) survival curve was used to determine the progression-free survival, disease-free survival, and overall survival status between the GPD2 low-expression and high-expression groups.

### Cell culture

The LUAD cell lines A549 and H1299 were obtained from the typical culture preservation committee of the Chinese academy of sciences (Shanghai, China). A549 cell was cultured in F12 medium (Hyclone, USA) and H1299 cell was cultured with RPMI-1640 medium (Hyclone, USA), which contained 10% fetal bovine serum (Hyclone, USA) and 1% penicillin-streptomycin (Hyclone, USA) at 37℃ with 5% CO2.

### CCK8 assay and cell status

Cell viability was detected by CCK8 assay. CCK8 assay is widely used in cell proliferation and cytotoxicity of rapid, high sensitivity, non-radioactive colorimetric detection kit. A total of 2*10^^3^ cells were planted in 96-well culture plates. Different concentrations of GPD2 target inhibitor-KM04416 (MedChemExpress, USA) were applied to cell co-culture. After 72 h of cell co-culture, 10 µl CCK8 solution was added into per well for 2 h culture. After that, the absorbance values of cells were measured at 450 nm with an enzyme microplate reader. The absorbance values represent of cell viability. In cell state detection, A total of 1*10^^5^ cells were planted in 24-well plates, and co-cultured with DMSO or KM04416 for 48 h. Then, wash the medium, and microphotography.

### 5-ethynyl-20-deoxyuridine (EdU assay)

The proliferation ability of A549 and H1299 cells was detected by BeyoClick™ EdU Cell Proliferation Kit with Alexa Fluor 488 (Beyotime, China). Cells (5*10^^4^) were plated in 24-well plates and cultured with DMSO or KM04416 for 24 h, and then the medium was replaced by 500 µL 10 µM EdU solution for 2 h in 37℃. 4% of paraformaldehyde was used to fix cells, and permeabilized by 0.3% Triton-100 for 15 min at room temperature. Azide 488 stained the proliferation cells, and Hoechst stained the nuclear of all cells.

### Colony formation

Cells were seeded in six-well plates with 1*10^^3^ cells per well, and cultured with DMSO or KM04416 for 7 days. Cells were fixed by polyformaldehyde, and stained by crystal violet.

### Migration and invasion assays

Transwell assay were used to detect cell migration and invasion abilities. In the invasion assay, Matrigel was used to cover the chamber overnight before the experiment. A density of 5*10^^4^ A549 and H1299 cells pretreated by DMSO or KM04416 was added in the upper chamber with 200 µl serum-free medium. Meanwhile, the lower chamber was added with 500 µl complete medium. After 24 h of incubation, 4% paraformaldehyde was used to fix cells and stained by crystal violet.

### Flow cytometric analysis

Cell cycle of A549 and H1299 cells was detected by Cell Cycle and Apoptosis Analysis Kit (Beyotime, China). Cell apoptosis of A549 and H1299 cells was evaluated by annexin V-FITC Apoptosis Detection Reagent (Beyotime, China). The experiments were conducted according manufacturer’s introduction.

### Immune infiltration

Our study investigated the relationships between GPD2 and lymphocyte and Major Histocompatibility Complex (MHC) molecule in LUAD by using Spearman’s correlation analysis in TISIDB website (http://cis.hku.hk/TISIDB/index.php) [13, 14]. TISIDB is a web portal for tumor and immune system interaction, which integrates multiple heterogeneous data types. When we explore the relationships between GPD2 expression and immune status, we only need to input GPD2, then the website will analyze the relationships between GPD2 expression and immune infiltration in LUAD microenvironment.

### Statistically analysis

The Wilcoxon test analyzed the GPD2 expression in LUAD and para-cancer tissue of LUAD. The prognosis predictive values of GPD2 in LUAD were analyzed by Kaplan-Meier analysis. Differences between DMSO and KM04416 treatment were evaluated by Student’s t-test. A p-value less than 0.05 was considered statistical significance.

## Results

### The GPD2 expression in LUAD tissues and survival status

**Fig. 1:**
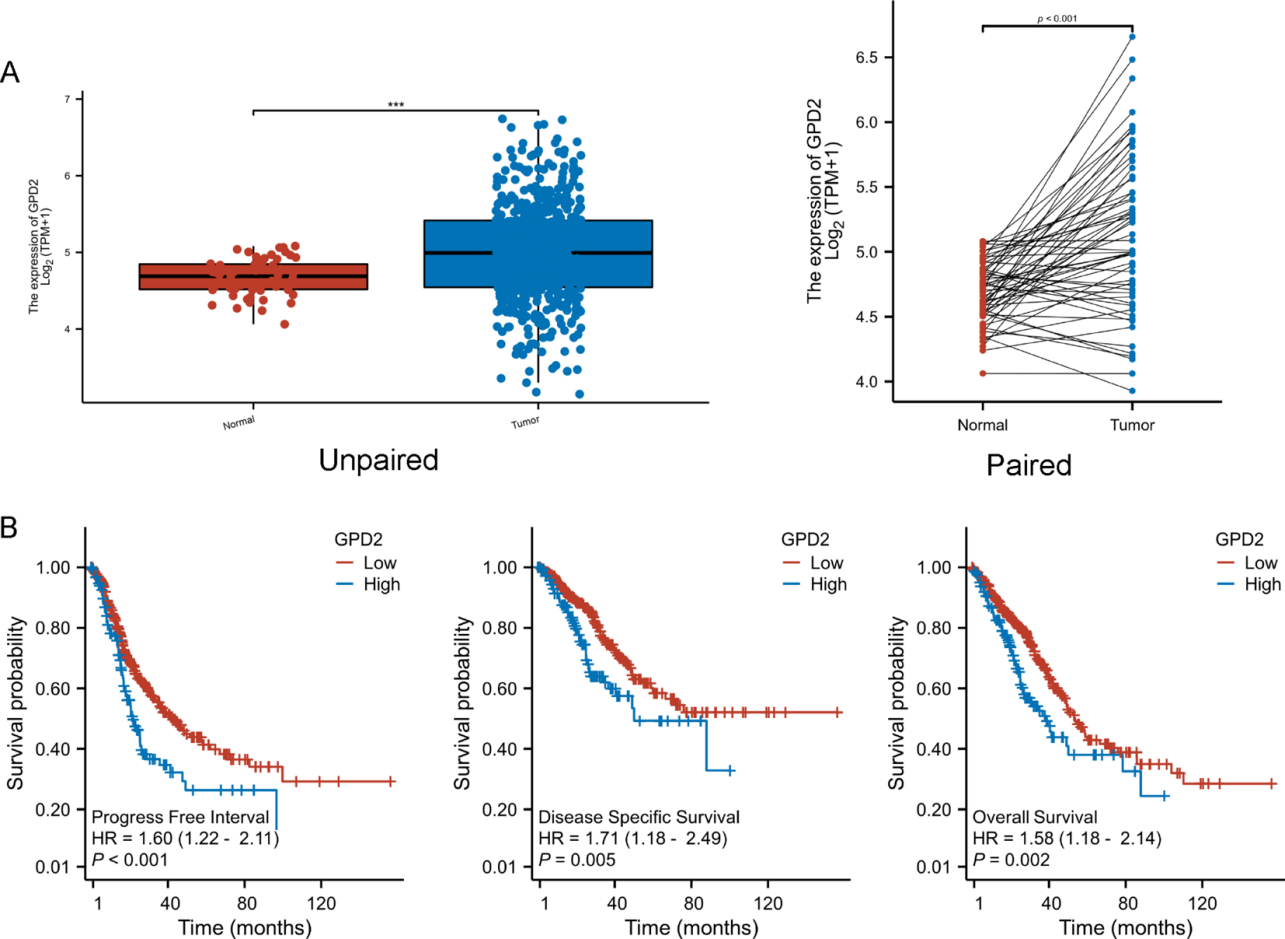
The expression levels of GPD2 in LUAD and survival status. (**A**) The expression levels of GPD2 in unpaired and paired LUAD tissues and normal lung tissues; (**B**) High expression of GPD2 is closely related to poor progress free interval, disease-specific survival and overall survival in LUAD

The GPD2 expression in LUAD and compared normal tissues were analyzed in the TCGA-LUAD database. As shown in Fig. 1A, the results confirmed that GPD2 is high expressed in LUAD tissues compared to normal tissues in paired or unpaired group. In Fig. 1B, LUAD group in TCGA-LUAD database is further divided into high expression and low expression, and analyzed the Progress Free Interval, Disease Specific Survival and Overall Survival in high expression and low expression of GPD2 in LUAD patients from TCGA-LUAD database which containing the clinical data of these patients by Kaplan-Meier (KM) survival analysis, and the results showed that GPD2 expression is associated to poor outcome in LUAD patients.

### KM04416 structure and cell viability, state changes after KM04416 treatment

**Fig. 2:**
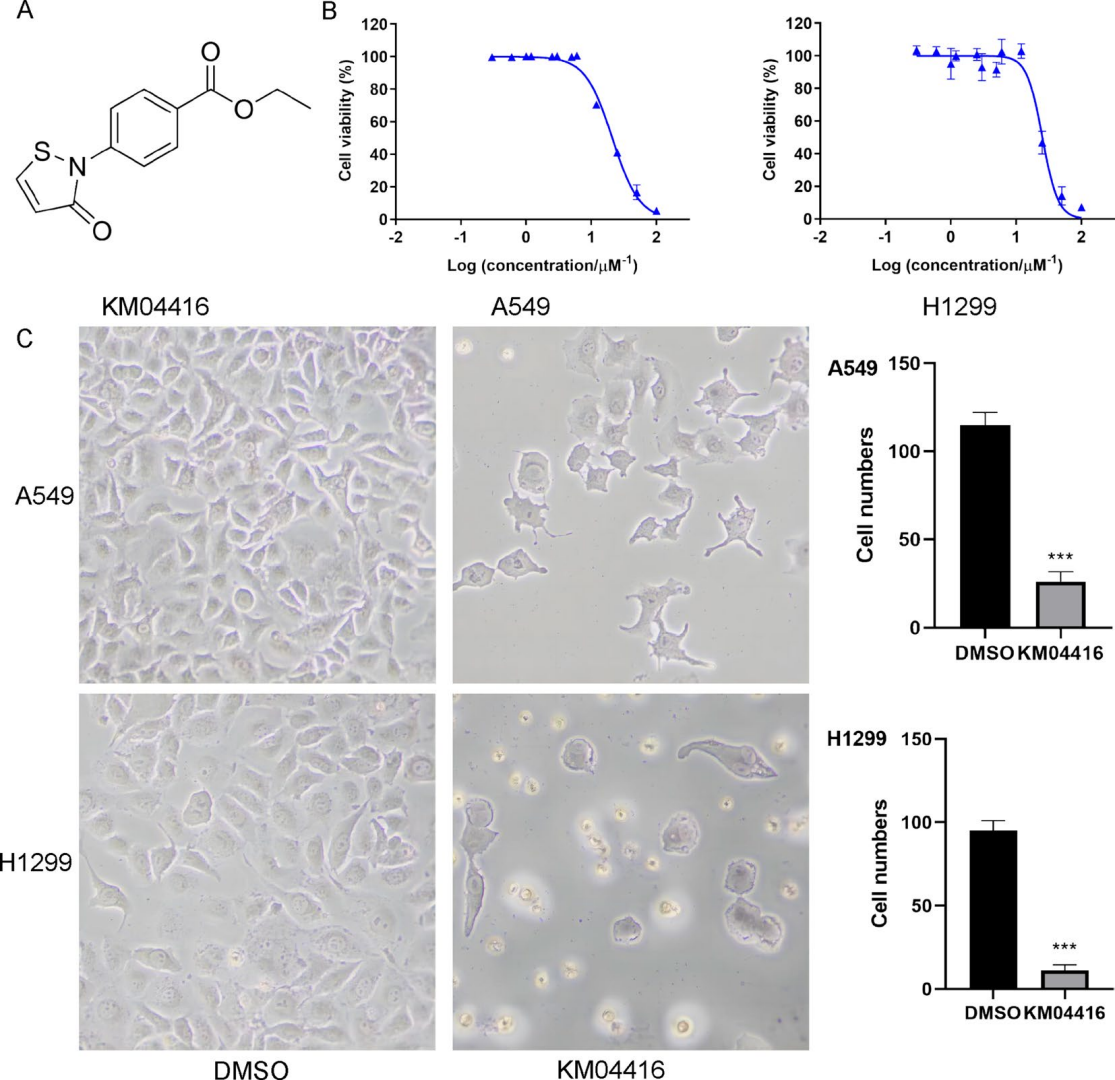
KM04416 structure and cell viability, state change after KM04416 treatment. (**A**) The molecular structure of KM04416; (**B**) Cell viability was decreased with the increased concentrations of KM04416 in A549 and H1299 cells; (**C**) The state of A549 and H1299 cells were remarkably suppressed after treated by KM04416

As shown in Fig. 2A, the structure of KM04416 was demonstrated. Different concentrations of KM04416 were used to co-culture with A549 and H1299 cells, and cell viability changes were detected by CCK-8 assay. The results found that cell viability was inhibited with the increased concentration of KM04416 (Fig. 2B). In addition, cell states were also suppressed in A549 and H1299 cells after KM04416 treatment (Fig. 2C).

### KM04416 suppressed the proliferation of LUAD cells

**Fig. 3:**
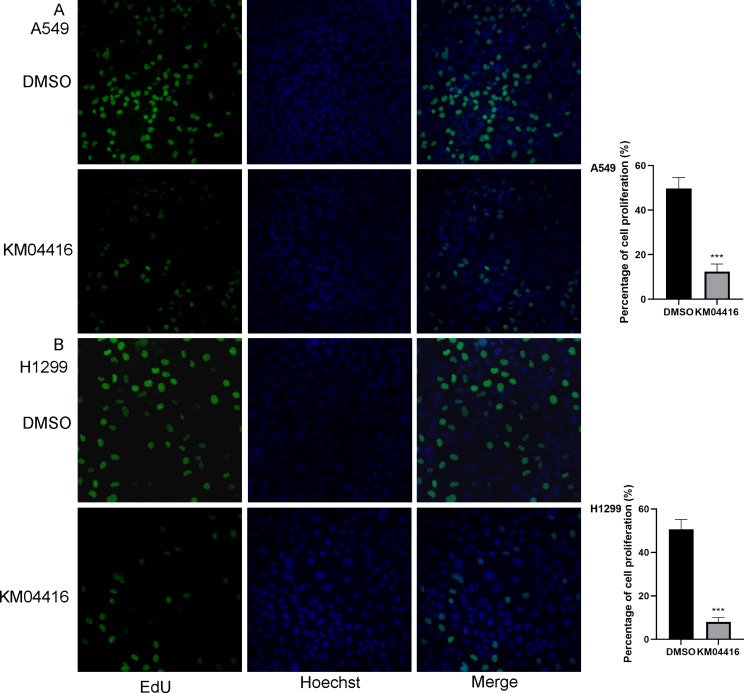
The proliferation ability changes of KM04416 treatment. (**A**) The treatment of KM04416 significantly inhibit the proliferation ability in the A549 cell; (**B**) The proliferation ability in the H1299 cell was suppressed by the KM04416 treatment

**Fig. 4:**
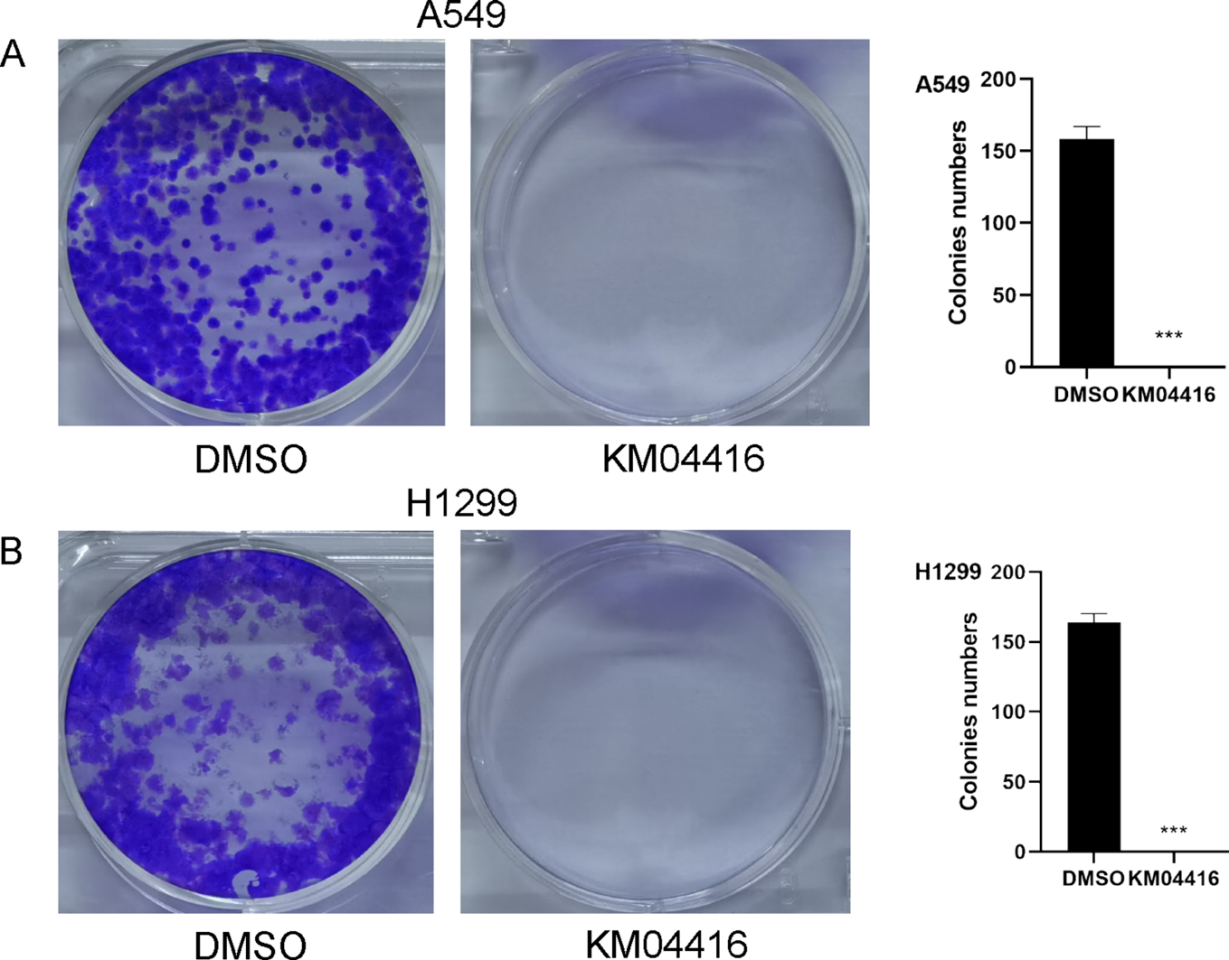
The differences of colony formation in DMSO or KM04416 groups. (**A**) The colony numbers in A549 cells were remarkably reduced after KM04416 treatment; (**B**) The treatment of KM04416 significantly decreased the colony numbers in the H1299 cell

EdU and colony formation experiments were used to investigate whether KM04416 affects the proliferation. The EdU experiment showed that cell proliferation rates are clearly reduced after KM04416 treatment (Fig. 3A, B). Meanwhile, the colony formation confirmed that the cell colony numbers was decreased in A549 and H1299 cells after treated by KM04416 (Fig. 4A, B).

### KM04416 suppressed the migration and invasion in LUAD cells

**Fig. 5:**
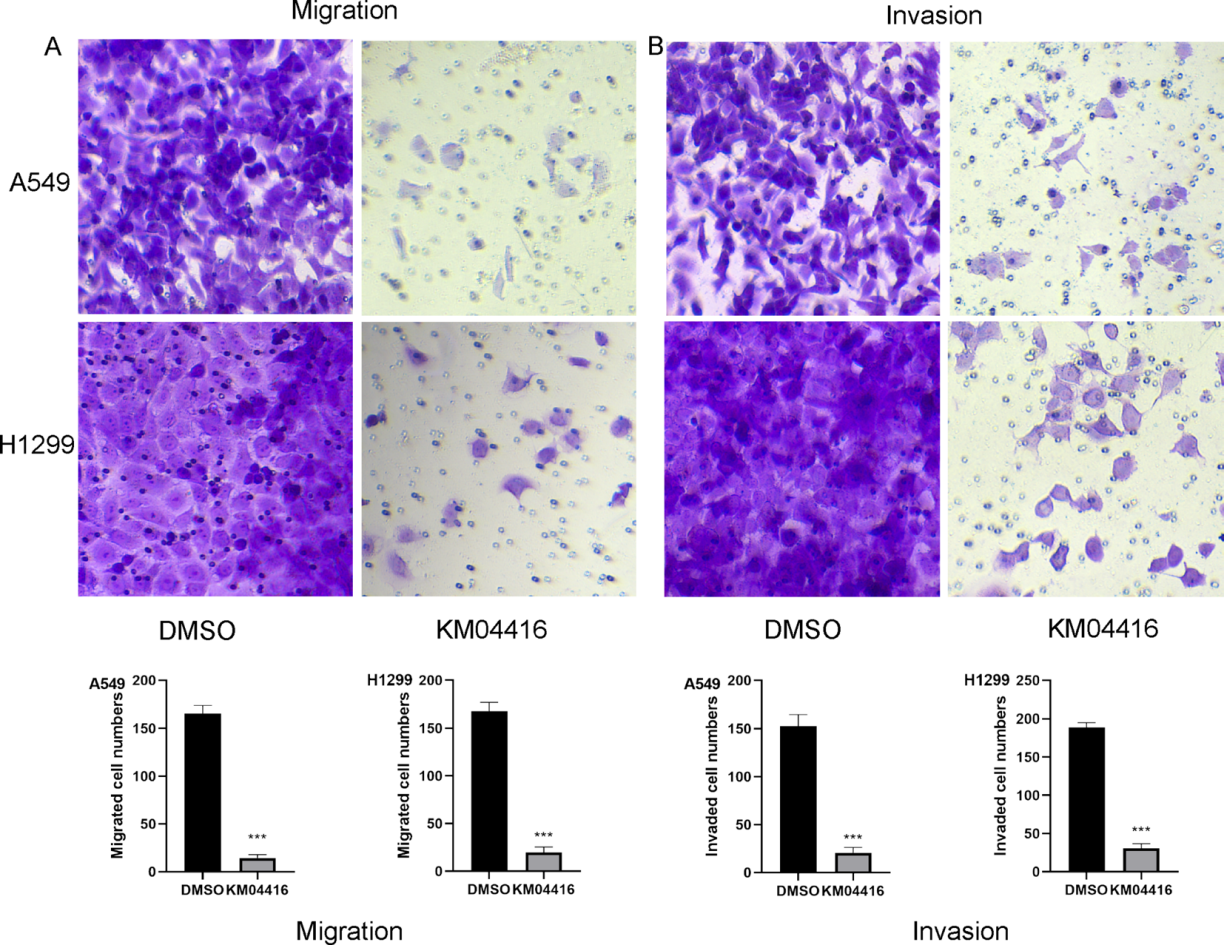
The migration and invasion abilities change in DMSO or KM04416 groups. (**A**) KM04416 treatment suppressed cell migration ability in A549 and H1299 cells; (**B**) KM04416 treatment suppressed cell invasion ability in A549 and H1299 cells

Transwell assay was applied to analyze whether inhibition of GPD2 by KM04416 affects the migration and invasion ability of A549 and H1299 cells. The results showed that KM04416 can remarkably suppress cell migration and invasion abilities in A549 and H1299 cells (Fig. 5A, B).

### KM04416 leads to cell cycle arrest and promote cell apoptosis in LUAD cells

**Fig. 6:**
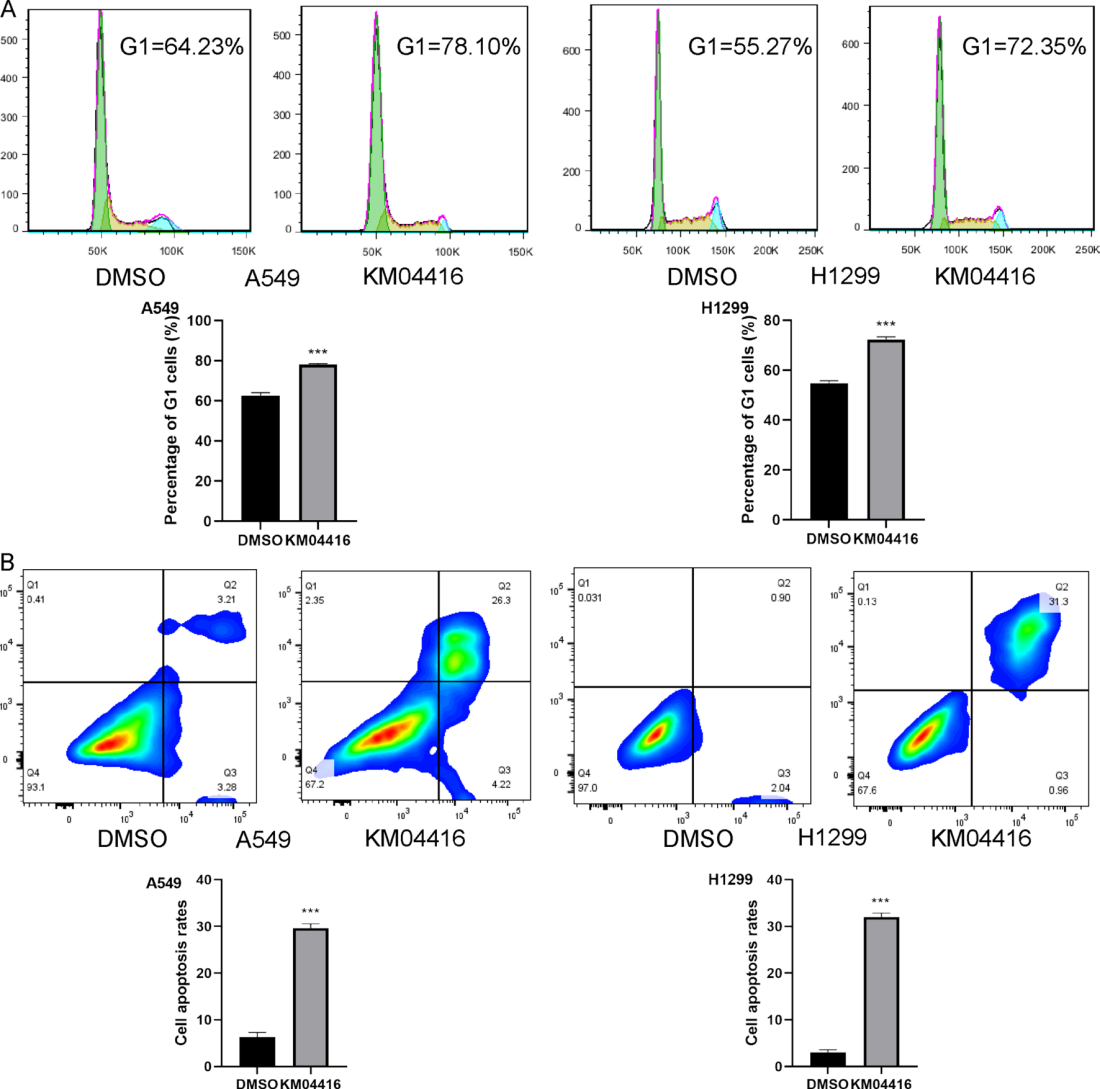
Cell cycle and cell apoptosis changes in DMSO or KM04416 groups. (**A**) KM04416 treatment leads to cell replication stagnating in G1 synthesis in A549 and H1299 cells; (**B**) KM04416 treatment promotes the percentage of apoptotic cells in A549 and H1299 cells

Through flow cytometry assay, we analyzed the cell cycle and cell apoptosis variation in A549 and H1299 cells treated by DMSO or KM04416. The cell cycle results showed that cell replication stagnated in G1 synthesis (early stage of DNA synthesis) (Fig. 6A). The apoptosis results found that KM04416 treatment can remarkably increase the percentage of apoptotic cells (Fig. 6B).

### GPD2 expression and immune infiltration in LUAD

**Fig. 7:**
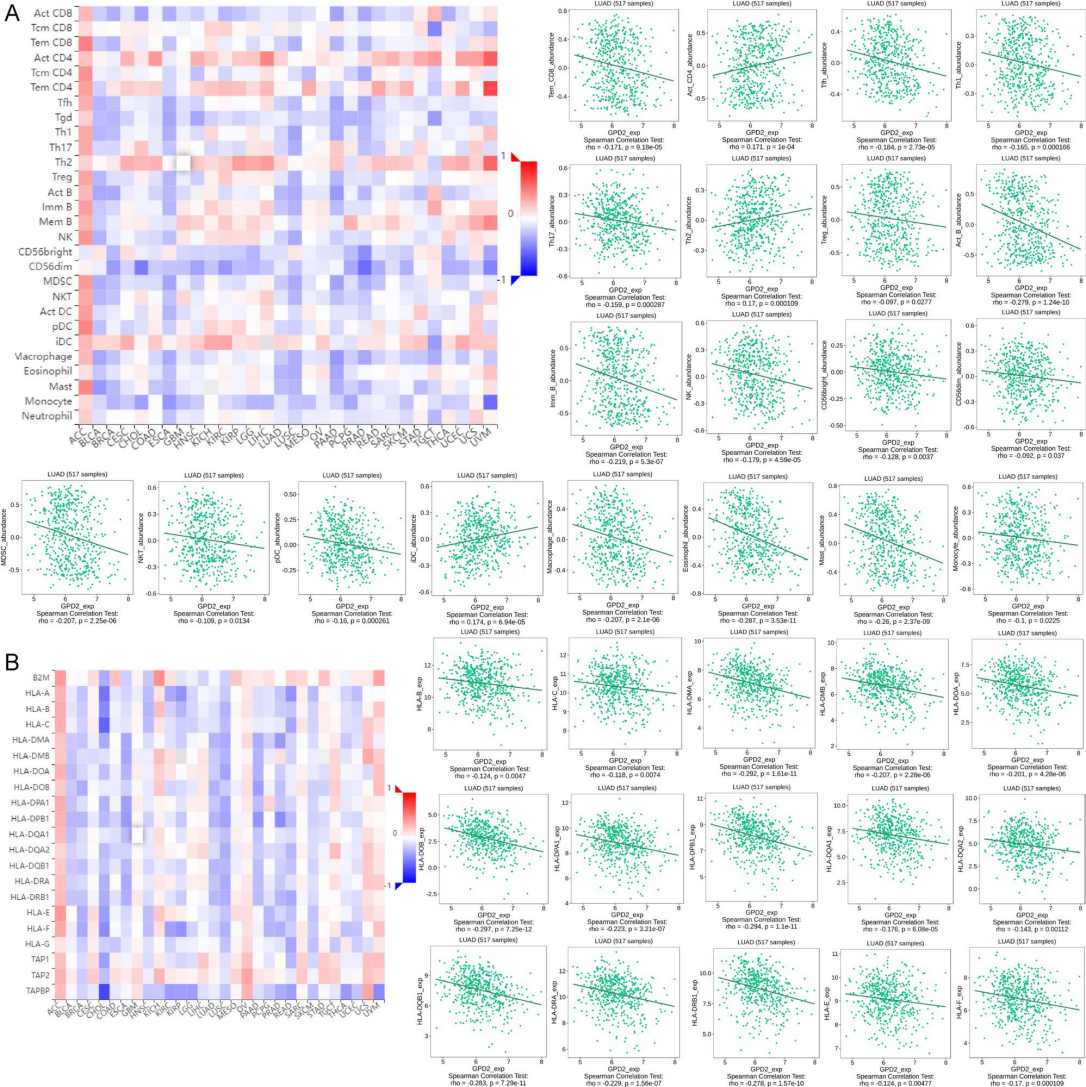
The correlations between GPD2 expression and tumor-infiltration lymphocytes, MHC molecular in LUAD based on the TISIB database. (**A**) Low expression of GPD2 relates to high infiltration of most immune cells, containing Tem_CD8, Tfh, Th1, Th17, Treg, Act_B, Imm_B, NK, CD56bright, CD56dim, MDSC, NKT, pDC, Macropahe, Eosinophil, Mast, Monocyte. Meanwhile, low expression of GPD2 relates to low expression of Act_CD4, Th2, iDC; (**B**) Low expression of GPD2 associate with high expression of HLA-B, HLA-C, HLA-DMA, HLA-DMB, HLA-DOA, HLA-DOB, HLA-DPA1, HLA-DPB1, HLA-DQA1, HLA-DQA2, HLA-DQB1, HLA-DRA, HLA-DRB1, HLA-E, HLA-F in LUAD

The TISIDB website applied to analyze the associations between GPD2 expression and immune infiltration levels in LUAD. The results found that low GPD2 expression relates to high infiltration of most immune cells, containing Tem_CD8, Tfh, Th1, Th17, Treg, Act_B, Imm_B, NK, CD56bright, CD56dim, MDSC, NKT, pDC, Macrophage, Eosinophil, Mast, Monocyte. Meanwhile, low expression of GPD2 relate to low expression of Act_CD4, Th2, iDC. Furthermore, we explored the relations between GPD2 expression and MHC molecule which found low GPD2 expression associate with high expression of HLA-B, HLA-C, HLA-DMA, HLA-DMB, HLA-DOA, HLA-DOB, HLA-DPA1, HLA-DPB1, HLA-DQA1, HLA-DQA2, HLA-DQB1, HLA-DRA, HLA-DRB1, HLA-E, HLA-F (Fig. 7).

## Discussion

LUAD presented first place in both morbidity and mortality in human cancers in recent decades, and posed a significant threat to human security [15, 16]. Due to the progression of immunotherapy and genetic target therapy in LUAD group, the survival outcome of LUAD patients have improved. However, most parts of LUAD patients are middle or late stages diagnosed, caused most LUAD patients initially present in the advanced stage and have a poor survival status [17]. Explore novel biomarkers which may predict the prognosis and have therapeutic values to further improve the survival rates of LUAD patients.

GPD2 is at the crossroads of several important cell metabolism processes, including triglyceride biosynthesis and phospholipid in lipid metabolism, gluconeogenesis in glucose metabolism, and electron transfer in mitochondrial [9]. The results of our study showed that GPD2 is up-regulated in LUAD compared to normal lung tissues. Low expression of GPD2 suggested a better survival outcome. Suppressed GPD2 by KM04416 can remarkably inhibit the progression of LUAD cell lines. The roles of GPD2 in other tumors have been explored. The inhibition of GPD2 is related to a reduction in oxidative phosphorylation, which has a negative role on thyroid cancer cell growth [18]. Meanwhile, Rauchova et al. [19] found that inhibition of GPD2 plays a role in α-Tocopheryl succinate-induced growth suppression in neoplastic cells. In prostate cancer, the promote GPD2 expression may result in the progression of the cancer through a highly glycolytic environment induce the overall increase in ROS generation [20]. In general, most studies have shown that high expression of GPD2 promotes tumor progression, and inhibiting GPD2 expression has potential therapeutic effect, which is consistent with our findings.

The tumor microenvironment (TME) is a complex integrated system composed mainly of tumor cells, inflammatory cells, nearby interstitial tissue, cancer-associated fibroblasts, and around immune cells [21, 22]. In our study, we analyzed the GPD2 expression and immune cell infiltration results in the TME of LUAD and found that low GPD2 expression associate with high infiltration of most immune cells, containing Tem_CD8, Tfh, Th1, Th17, Treg, Act_B, Imm_B, NK, CD56bright, CD56dim, MDSC, NKT, pDC, Macrophage, Eosinophil, Mast, Monocyte. Meanwhile, low GPD2 expression associated with low expression of Act_CD4, Th2, iDC. Furthermore, we explored the relations between GPD2 expression and MHC molecule which found that low expression of GPD2 associate with high expression of HLA-B, HLA-C, HLA-DMA, HLA-DMB, HLA-DOA, HLA-DOB, HLA-DPA1, HLA-DPB1, HLA-DQA1, HLA-DQA2, HLA-DQB1, HLA-DRA, HLA-DRB1, HLA-E, HLA-F. Accumulating evidence confirmed that the status of TME is closely associated with lung cancer progression, metastasis, and chemotherapy [23, 24]. The immune cell infiltration status of TME is related to the clinical outcome of lung cancer patients [25]. The most crucial role of MHC molecules is to participate in antigen presentation. MHC molecules bind to antigenic peptides through their peptide binding slots and present them on the cell surface for T cells to recognize and trigger an immune response [26]. Lu e tal. [27] found that inhibiting GPD2 in glioma cells can blockage intra-tumoral macrophage recruitment, and suggest possible treatment strategies for glioma patients. The study also confirmed that GPD2 expression is related to immune response according to mass spectrometry base proteomics profiling of human monocytes [28]. Therefore, GPD2 may plays functions through regulating immune cell infiltration in LUAD.

## Conclusions

In general, our present study found that GPD2 is overexpressed in LUAD, and the expression of GPD2 can be an independent prognosis predictor in LUAD. Suppressed GPD2 by target inhibitor KM04416 can restrain LUAD progression. The mechanism analysis suggested that GPD2 plays functions through regulating immune cell infiltration.

## Data Availability

No datasets were generated or analysed during the current study.
